# 494. Enteric bacteria are immune-reactive in patients with Crohn's disease with extraintestinal manifestations

**DOI:** 10.1093/ofid/ofac492.552

**Published:** 2022-12-15

**Authors:** Grace A Maldarelli, Maeva Metz, Gabriela Funez-dePagnier, Alexa Lavergne, Daniel Lai, Adrienne Chang, Fardina Malik, Iwijn de Vlaminck, Randy S Longman

**Affiliations:** NewYork Presbyterian - Weill Cornell Medicine, New York, New York; Jill Roberts Institute for IBD Research, Weill Cornell Medicine, New York, New York; Jill Roberts Center for IBD, Division of Gastroenterology and Hepatology, Department of Medicine, Weill Cornell Medicine, New York, New York; Jill Roberts Center for IBD, Division of Gastroenterology and Hepatology, Department of Medicine, Weill Cornell Medicine, New York, New York; Weill Cornell Medical College – Cornell University, New York, New York; Meinig School of Biomedical Engineering – Cornell University, Ithaca, New York; NYU Grossman School of Medicine, Division of Rheumatology, New York, New York; Meinig School of Biomedical Engineering – Cornell University, Ithaca, New York; Jill Roberts Center for IBD, Division of Gastroenterology and Hepatology, Department of Medicine, Weill Cornell Medicine, New York, New York

## Abstract

**Background:**

Bacterial constituents of the intestinal microbiota can contribute to local and systemic inflammatory diseases. Crohn's disease (CD), a type of inflammatory bowel disease, can result in extraintestinal manifestations (EIM) including peripheral (CD-SpA) and axial spondyloarthritis (CD-AxSpA). Microbial contributors to CD pathogenesis can be identified by flow cytometric sorting and 16S sequencing of bacteria recognized by mucosal IgA. We expand this technique by incubating fecal samples with autologous sera, capturing bacterial species recognized by circulating IgG in a process called IgG-seq. We hypothesize that these systemically-recognized enteric bacteria are linked to development of CD-EIM.

**Methods:**

Fecal samples from individuals with CD were sorted into IgG-positive and negative populations. DNA was extracted from each population, 16S sequencing performed, and sequences processed with QIIME2. Intestinal coating index (ICI) was calculated at the genus level. Serum cell-free DNA sequencing (cfDNA) was performed for a subset of samples.

**Results:**

IgG-seq was conducted on 86 CD, 41 CD-SpA, and 16 CD-AxSpA samples. PCoA analysis demonstrates significant differences in microbiome composition in the three groups (P = 0.013, PERMANOVA). Relative abundances of *Escherichia-Shigella* and *Ruminococcus* are positively correlated with joint disease activity. Analysis of IgG ICI for genera present in > 10% of patients demonstrates overrepresentation of *Ruminococcus, Escherichia-Shigella,* and *Bacteroides* in IgG-recognized fractions. IgG recognition of bacteria does not cluster specifically by CD or EIM severity. cfDNA sequencing demonstrates no *Ruminococcus* DNA in serum, whereas *E. coli* DNA was detected in multiple patient sera.

**Conclusion:**

IgG-seq is a robust method for identifying immune-reactive enteric bacteria; our results highlight the link between immune response to enteric bacterial genera, such as *Escherichia-Shigella* and *Ruminococcus*, and joint disease activity in CD-SpA and CD-AxSpA. While Escherichia-Shigella may induce immunity by breaching the mucosal barrier, the absence of *Ruminococcus* in serum cfDNA suggests immune activation at barrier sites. Further studies are needed to characterize the strain-specific features that underlie our findings.

**Disclosures:**

**Fardina Malik, MD**, Pfizer: Advisor/Consultant **Iwijn de Vlaminck, PhD**, GenDX: Advisor/Consultant|GenDX: Board Member|Kanvas Biosciences: Board Member|Kanvas Biosciences: Ownership Interest|Karius Inc.: Board Member|Karius Inc.: Ownership Interest|Viracor Eurofins: Advisor/Consultant **Randy S. Longman, MD, PhD**, Pfizer: Advisor/Consultant.

